# Supplementary material for: Improving the Detection of Epidemic Clones in Candida parapsilosis Outbreaks by Combining MALDI-TOF Mass Spectrometry and Deep Learning Approaches
Source: Microorganisms. 2023 Apr 20;11(4):1071. doi: 10.3390/microorganisms11041071 (PMC10146746; doi:10.3390/microorganisms11041071)
Supplement: Supplementary file 1 [file microorganisms-11-01071-s001.zip › microorganisms-2313491-supplementary.pdf]

Table S1: Origin and characteristics of the 96 isolates used for this study.

CODE	CLONALITY	SUSCEPTIBILITY	Origin	ISOLATION DATE	PUBLISHED IN	allelic profile (3A-3A; 3B-3B; 3C-3C; 6A-6A; 6B-6B; 6C-6C)
101		0,75	PSL	oct-18	FEKKAR et al.	(28-35 ; 31-36 ; 44-45 ; 13-17 ; 7-7 ; 7-7)
102	clone set	>256	PSL	oct-18	FEKKAR et al.	(28-28 ; 49-82 ; 48-51 ; 8-8 ; 7-7 ; 7-7)
103		0,19	PSL	oct-18	FEKKAR et al.	(28-28 ; 33-36 ; 32-37 ; 12-12 ; 7-7 ; 7-7)
104		1	PSL	oct-18	FEKKAR et al.	(27-28 ; 46-47 ; 38-39 ; 8-8 ; 7-7 ; 7-7)
105		0,38	PSL	oct-18	FEKKAR et al.	(27-28 ; 31-31 ; 34-34 ; 12-12 ; 7-7 ; 7-7)
106	clone set	>256	PSL	oct-18	FEKKAR et al.	(28-28 ; 49-82 ; 48-51 ; 8-8 ; 7-7 ; 7-7)
107	clone set	>256	PSL	oct-18	FEKKAR et al.	(28-28 ; 49-82 ; 48-51 ; 8-8 ; 7-7 ; 7-7)
108		0,38	PSL	nov-18	FEKKAR et al.	(28-35 ; 31-36 ; 44-45 ; 13-17 ; 7-7 ; 7-7)
109		0,75	PSL	nov-18	FEKKAR et al.	(28-29 ; 45-46 ; 46-46 ; 8-8 ; 7-7 ; 7-7)
110		1,5	PSL	nov-18	FEKKAR et al.	(21-35 ; 30-31 ; 36-37 ; 13-13 ; 9-9 ; 7-7)
112		0,19	PSL	nov-18	FEKKAR et al.	(22-29 ; 52-52 ; 36-56 ; 12-13 ; 7-7 ; 7-7)
116		0,19	PSL	nov-18	FEKKAR et al.	(28-29 ; 44-47 ; 39-39 ; 13-17 ; 7-7 ; 7-7)
117		0,5	PSL	nov-18	FEKKAR et al.	(28-28 ; 42-49 ; 30-39 ; 8-13 ; 7-7 ; 7-7)
118		1,5	PSL	nov-18	FEKKAR et al.	(29-29 ; 52-52 ; 44-51 ; 12-12 ; 7-7 ; 7-7)
119		>256	PSL	déc-18	FEKKAR et al.	(28-30 ; 59-63 ; 43-43 ; 12-12 ; 7-7 ; 7-7)
121		0,75	PSL	déc-18	FEKKAR et al.	(28-29 ; 36-47 ; 39-39 ; 13-17 ; 7-7 ; 7-7)
123		1	PSL	déc-18	FEKKAR et al.	(28-28 ; 46-46 ; 39-46 ; 8-8 ; 7-7 ; 7-7)
124		1,5	PSL	déc-18	FEKKAR et al.	(28-29 ; 38-43 ; 42-47 ; 10-13 ; 7-7 ; 7-7)
126	clone set	>256	PSL	déc-18	FEKKAR et al.	(28-28 ; 49-82 ; 48-51 ; 8-8 ; 7-7 ; 7-7)
127		0,75	PSL	janv-19	FEKKAR et al.	(24-28 ; 47-52 ; 39-39 ; 8-8 ; 7-7 ; 7-7)
128		1	PSL	janv-19	FEKKAR et al.	(21-35 ; 31-31 ; 37-37 ; 13-13 ; 7-9 ; 7-7)
129		1	PSL	janv-19	FEKKAR et al.	(27-28 ; 69-69 ; 40-46 ; 8-8 ; 7-7 ; 7-7)
130		0,5	PSL	janv-19	FEKKAR et al.	(29-29 ; 20-32 ; 31-31 ; 12-12 ; 10-12 ; 7-7)
131		0,25	PSL	janv-19	FEKKAR et al.	(17-28 ; 32-36 ; 43-43 ; 12-12 ; 7-7 ; 7-7)
133		0,25	PSL	janv-19	FEKKAR et al.	(17-28 ; 32-36 ; 42-52 ; 12-12 ; 7-7 ; 7-7)
134		1	PSL	janv-19	FEKKAR et al.	(28-29 ; 33-33 ; 36-38 ; 12-12 ; 10-12 ; 7-7)
135		1,5	PSL	janv-19	FEKKAR et al.	(27-29 ; 36-47 ; 39-39 ; 13-17 ; 7-7 ; 7-7)
138		1,5	PSL	janv-19	FEKKAR et al.	(25-28 ; 48-48 ; 39-39 ; 8-8 ; 7-7 ; 7-7)
139		1	PSL	févr-19	FEKKAR et al.	(28-29 ; 55-55 ; 53-57 ; 8-8 ; 7-7 ; 7-7)
143		0,5	PSL	févr-19	FEKKAR et al.	(27-27 ; 47-47 ; 39-39 ; 8-8 ; 7-7 ; 7-7)
146		0,75	PSL	févr-19	FEKKAR et al.	(28-28 ; 55-55 ; 53-53 ; 8-8 ; 7-7 ; 7-7)
147		0,75	PSL	févr-19	FEKKAR et al.	(28-29 ; 36-36 ; 39-39 ; 17-17 ; 7-7 ; 7-7)
148		0,5	PSL	mars-19	FEKKAR et al.	(28-28 ; 47-47 ; 39-39 ; 7-8 ; 7-7 ; 7-7)
149		1	PSL	mars-19	FEKKAR et al.	(27-29 ; 36-47 ; 38-38 ; 17-17 ; 7-7 ; 7-7)
150		1,5	PSL	mars-19	FEKKAR et al.	(28-28 ; 36-36 ; 47-47 ; 13-14 ; 9-9 ; 7-7)
151	clone set	>256	PSL	mars-19	FEKKAR et al.	(28-28 ; 49-82 ; 48-51 ; 8-8 ; 7-7 ; 7-7)
152		0,5	PSL	mars-19	FEKKAR et al.	(28-28 ; 47-47 ; 38-38 ; 7-7 ; 7-7 ; 7-7)
153		0,5	PSL	avr-19	FEKKAR et al.	(28-28 ; 30-30 ; 33-33 ; 12-12 ; 9-9 ; 8-8)
154		0,25	PSL	avr-19	FEKKAR et al.	(28-28 ; 46-48 ; 39-39 ; 8-8 ; 7-7 ; 7-7)
155		0,5	PSL	avr-19	FEKKAR et al.	(29-29 ; 20-32 ; 31-36 ; 12-12 ; 10-12 ; 7-7)
156		1	PSL	avr-19	FEKKAR et al.	(22-29 ; 52-52 ; 38-55 ; 12-13 ; 7-7 ; 7-7)
157		1	PSL	mai-19	FEKKAR et al.	(28-28 ; 32-32 ; 39-46 ; 13-13 ; 7-10 ; 7-7)
158		0,25	PSL	mai-19	FEKKAR et al.	(28-28 ; 36-36 ; 40-40 ; 13-17 ; 7-7 ; 7-7)
162		1,75	PSL	juin-19	FEKKAR et al.	(28-28 ; 47-47 ; 39-39 ; 13-17 ; 7-7 ; 7-7)
172		24	PSL	juil-19	FEKKAR et al.	(28-28 ; 45-45 ; 46-46 ; 8-8 ; 7-7 ; 7-7)
177		3	PSL	juil-19	FEKKAR et al.	(22-27 ; 52-62 ; 38-49 ; 12-12 ; 7-13 ; 7-7)
201	clone set	>256	PSL	août-19	FEKKAR et al.	(28-28 ; 49-82 ; 48-51 ; 8-8 ; 7-7 ; 7-7)
208		0,25	PSL	août-19	FEKKAR et al.	(27-29 ; 31-31 ; 36-36 ; 12-12 ; 7-7 ; 7-7)
209		0,25	PSL	août-19	FEKKAR et al.	(28-28 ; 49-56 ; 37-37 ; 12-12 ; 7-7 ; 7-7)
211	clone set	>256	PSL	sept-19	FEKKAR et al.	(28-28 ; 49-82 ; 48-51 ; 8-8 ; 7-7 ; 7-7)
214		0,75	PSL	sept-19	FEKKAR et al.	(22-29 ; 52-52 ; 36-36 ; 12-12 ; 7-7 ; 7-7)
220		1	PSL	sept-19	FEKKAR et al.	(29-29 ; 52-52 ; 44-51 ; 12-12 ; 7-7 ; 7-7)
225	clone set	>256	PSL	oct-19	FEKKAR et al.	(28-28 ; 49-82 ; 48-51 ; 8-8 ; 7-7 ; 7-7)
227		0,38	PSL	oct-19	FEKKAR et al.	(27-28 ; 31-31 ; 33-42 ; 11-12 ; 7-7 ; 7-7)
228		48	PSL	oct-19	FEKKAR et al.	(29-30 ; 52-52 ; 44-51 ; 12-32 ; 7-7 ; 7-7)
257	clone set	>256	PSL	févr-20	unpublished	(28-28 ; 49-75 ; 48-51 ; 8-8 ; 7-7 ; 7-7)
259	clone set	>256	PSL	févr-20	unpublished	(28-28 ; 49-82 ; 48-51 ; 8-8 ; 7-7 ; 7-7)
261	clone set	>256	PSL	févr-20	unpublished	(28-28 ; 49-82 ; 48-51 ; 8-8 ; 7-7 ; 7-7)
274	clone set	>256	PSL	avr-20	unpublished	(28-28 ; 49-82 ; 48-51 ; 8-8 ; 7-7 ; 7-7)
280	clone set	>256	PSL	avr-20	unpublished	(28-28 ; 49-75 ; 48-51 ; 8-8 ; 7-7 ; 7-7)
281		0,125	PSL	avr-20	FEKKAR et al.	(28-28 ; 62-62 ; 49-49 ; 12-14 ; 7-14 ; 7-7)
303	clone set	>256	PSL	avr-20	unpublished	(28-28 ; 49-82 ; 48-51 ; 8-8 ; 7-7 ; 7-7)
304	clone set	>256	PSL	avr-20	unpublished	(28-28 ; 49-82 ; 48-51 ; 8-8 ; 7-7 ; 7-7)
306		128	PSL	mai-20	unpublished	(29-29 ; 36-47 ; 39-39 ; 13-17 ; 7-7 ; 7-7)
307	clone set	>256	PSL	avr-20	unpublished	(28-28 ; 49-76 ; 48-51 ; 8-8 ; 7-7 ; 7-7)
312	clone set	>256	PSL	mai-20	unpublished	(28-28 ; 49-75 ; 48-51 ; 8-8 ; 7-7 ; 7-7)
322	clone set	>256	PSL	juin-20	unpublished	(28-28 ; 49-75 ; 48-51 ; 8-8 ; 7-7 ; 7-7)
324	clone set	>256	PSL	juin-20	unpublished	(28-28 ; 49-82 ; 48-51 ; 8-8 ; 7-7 ; 7-7)
329	clone set	16	PSL	juil-20	unpublished	(28-28 ; 46-82 ; 48-51 ; 8-8 ; 7-7 ; 7-7)
330		4	PSL	juil-20	unpublished	(18-29 ; 31-51 ; 14-48 ; 7-9 ; 7-9 ; 7-7)
337		>256	PSL	juil-20	unpublished	(28-29 ; 28-28 ; 50-50 ; 13-33 ; 7-7 ; 7-7)
339		96	PSL	juil-20	unpublished	(28-29 ; 32-45 ; 35-40 ; 12-32 ; 12-12 ; 7-7)
344	clone set	>256	PSL	août-20	unpublished	(28-28 ; 49-82 ; 48-51 ; 8-8 ; 7-7 ; 7-7)
349	clone set	>256	PSL	août-20	unpublished	(28-28 ; 49-82 ; 48-51 ; 8-8 ; 7-7 ; 7-7)

350	clone set	>256	PSL	août-20	unpublished	(28-28 ; 49-82 ; 48-51 ; 8-8 ; 7-7 ; 7-7)
360	clone set	>256	PSL	oct-20	unpublished	(28-28 ; 49-82 ; 48-51 ; 8-8 ; 7-7 ; 7-7)
361	clone set	>256	PSL	oct-20	unpublished	(28-28 ; 49-82 ; 48-51 ; 8-8 ; 7-7 ; 7-7)
BCHFR1	clone set	>256	BCH	mai-21	PRESENTE et al.	(28-28 ; 49-75 ; 48-51 ; 8-8 ; 7-7 ; 7-7)
BCHFR3	clone set	>256	BCH	mai-21	PRESENTE et al.	(28-28 ; 49-75 ; 48-51 ; 8-8 ; 7-7 ; 7-7)
BCHFR4	clone set	>256	BCH	juin-21	PRESENTE et al.	(28-28 ; 49-75 ; 48-51 ; 8-8 ; 7-7 ; 7-7)
BCHFR5	clone set	>256	BCH	juin-21	PRESENTE et al.	(28-28 ; 49-75 ; 48-51 ; 8-8 ; 7-7 ; 7-7)
BCHFR6	clone set	>256	BCH	juin-21	PRESENTE et al.	(28-28 ; 49-75 ; 48-51 ; 8-8 ; 7-7 ; 7-7)
BCHFR7	clone set	>256	BCH	juin-21	PRESENTE et al.	(28-28 ; 49-82 ; 48-51 ; 8-8 ; 7-7 ; 7-7)
BCHFR8	clone set	>256	BCH	juin-21	PRESENTE et al.	(28-28 ; 49-75 ; 48-51 ; 8-8 ; 7-7 ; 7-7)
BCHFR9	clone set	>256	BCH	juin-21	PRESENTE et al.	(28-28 ; 49-75 ; 48-51 ; 8-8 ; 7-7 ; 7-7)
BDXAZA		>256	Bordeaux	nov-21	unpublished	(28-29 ; 21-21 ; 39-39 ; 12-12 ; 10-12 ; 7-7)
BDXVIL		8	Bordeaux	nov-21	unpublished	(32-33 ; 31-31 ; 45-45 ; 13-13 ; 7-7 ; 7-7)
PARAR10		64	PSL	mars-12	FEKKAR et al.	(28-29 ; 36-59 ; 38-39 ; 18-18 ; 7-7 ; 7-7)
PARAR13	clone set	>256	PSL	août-18	FEKKAR et al.	(28-28 ; 49-82 ; 48-51 ; 8-8 ; 7-7 ; 7-7)
PARAR18	clone set	>256	PSL	nov-17	FEKKAR et al.	(28-28 ; 49-82 ; 48-51 ; 8-8 ; 7-7 ; 7-7)
PARAR19	clone set	>256	PSL	oct-18	FEKKAR et al.	(28-28 ; 49-82 ; 48-51 ; 8-8 ; 7-7 ; 7-7)
PARAR2		>256	PSL	nov-17	unpublished	(28-29 ; 36-56 ; 39-39 ; 13-17 ; 7-7 ; 7-7)
PARAR20		8	PSL	août-13	FEKKAR et al.	(28-32 ; 32-33 ; 39-45 ; 13-15 ; 7-7 ; 7-7)
PARAR4	clone set	>256	PSL	nov-17	FEKKAR et al.	(28-28 ; 49-82 ; 48-51 ; 8-8 ; 7-7 ; 7-7)
PARAR5	clone set	>256	PSL	janv-18	FEKKAR et al.	(28-28 ; 49-82 ; 48-51 ; 8-8 ; 7-7 ; 7-7)
PARAR8	clone set	>256	PSL	janv-18	FEKKAR et al.	(28-28 ; 49-82 ; 48-51 ; 8-8 ; 7-7 ; 7-7)
